# Age-related differences in BMD response during three years of denosumab treatment

**DOI:** 10.3389/fragi.2026.1892852

**Published:** 2026-08-13

**Authors:** Koji Ishikawa, Tomoyuki Asada, William Richardson, Choiselle Marius, Mahoko Ishikawa, Tuyet Nguyen, Philip Varnadore, Soji Tani, Peter Passias, Benjamin Aaron Alman

**Affiliations:** 1 Department of Orthopaedic Surgery, Duke University, Durham, NC, United States; 2 Department of Orthopedic Surgery, Tsukuba Memorial Hospital, Ibaraki, Japan; 3 Department of Orthopaedic Surgery, Showa Medical University, Tokyo, Japan

**Keywords:** aging, BMD, denosumab (anti-RANKL antibody), osteoporosis, treatment response

## Abstract

**Introduction:**

Denosumab increases bone mineral density and reduces fracture risk in patients with osteoporosis. However, whether BMD response to denosumab differs by age, particularly during longer term treatment, remains unclear. This study investigated the association between baseline age and BMD gain during 3 years of denosumab treatment in patients with osteoporosis.

**Methods:**

This retrospective study included patients with osteoporosis who were treated with denosumab. DXA-based BMD and bone turnover markers were followed for up to 3 years. Percent BMD gain from baseline was evaluated. The longitudinal association between baseline age and %BMD gain was assessed using multivariable linear mixed effects models for the lumbar spine and total hip. Analyses were performed in the treatment naive cohort and the overall cohort according to prior osteoporosis treatment status.

**Results:**

A total of 255 patients were included, of whom 110 were treatment naive. In multivariable linear mixed effects models, older baseline age was associated with smaller lumbar spine %BMD gain in the treatment naive cohort at both 1 and 3 years. Each 1 year increase in age was associated with a 0.187 percentage point lower lumbar spine %BMD gain at 1 year and a 0.293 percentage point lower gain at 3 years (1 year: β = −0.187, p = 0.006, 3 years: β = −0.293, p = 0.031). In contrast, baseline age was not significantly associated with total hip %BMD gain in the treatment naive cohort (1 year: β = −0.011, p = 0.826, 3 years: β = 0.028, p = 0.727). In the overall cohort, baseline age was not significantly associated with %BMD gain at either skeletal site.

**Conclusion:**

Older baseline age was associated with a modestly smaller lumbar spine BMD gain in treatment-naive patients, whereas no statistically significant age-related association was observed at the total hip. These findings indicate a modest, site-specific association between baseline age and BMD gain in treatment-naive patients.

## Introduction

Denosumab is a monoclonal antibody against receptor activator of nuclear factor κB ligand that suppresses osteoclast mediated bone resorption. In postmenopausal women with osteoporosis, denosumab increases bone mineral density and reduces the risk of fractures (Ferrari and Langdahl, 2023; Cummings et al., 2009). Long term extension studies have further shown progressive BMD gains at both the lumbar spine and hip for up to 10 years (Bone et al., 2017; Kendler et al., 2022). However, because the effects of denosumab are reversible after treatment interruption or discontinuation, denosumab initiation requires careful consideration of patient age, as age may influence the expected duration of treatment and long-term management strategy (Tsourdi et al., 2021).

Aging is accompanied by multiple skeletal changes, including reduced bone formation capacity, increased cortical porosity, and different patterns of trabecular and cortical bone loss across skeletal sites (Seeman, 2013; Khosla, 2013; Shanbhogue et al., 2016). These skeletal alterations are associated with changes in bone remodeling, with a shift toward osteoclast mediated resorption relative to bone formation (Raisz, 2005). As a result, the skeletal response to osteoporosis treatment may vary according to patient age and anatomical site. Although antiosteoporosis medications generally remain effective in older adults, previous studies have reported inconsistent findings regarding age related differences in BMD response and fracture outcomes (Schini et al., 2024; Marcus et al., 2003; McClung et al., 2001; Yoon et al., 2023; Jeong and Ha, 2020).

This question may be particularly relevant for denosumab because its therapeutic effects are mediated through direct inhibition of RANKL dependent osteoclast activity (Ferrari and Langdahl, 2023; Kendler et al., 2022). By suppressing osteoclast mediated bone resorption, denosumab produces sustained increases in BMD, but the magnitude of these gains may depend on the remodeling environment at treatment initiation. Therefore, age related differences in skeletal remodeling activity may influence the longitudinal BMD response to denosumab. Prior subgroup analyses of the FREEDOM trial have shown that denosumab reduces fracture risk in older women, including those aged 75 years or older, and improves BMD in this age group (McClung et al., 2012; Boonen et al., 2011). However, the association between baseline age and BMD gains during longer term denosumab treatment in routine clinical practice remains unclear. Clarifying this relationship may improve our understanding of age-related patterns in longitudinal BMD response during denosumab treatment. In this retrospective study, we investigated whether baseline age was associated with BMD response during 3 years of denosumab treatment in patients with osteoporosis.

## Materials and methods

### Study design and patient selection

This retrospective observational study included patients aged 50 years or older with primary osteoporosis in Japan who were treated with denosumab. The study was conducted in accordance with the principles of the Declaration of Helsinki. The requirement for written informed consent was waived because of the retrospective nature of the study. The data were mainly obtained from a pre-existing observational osteoporosis cohort established to follow patients who initiated osteoporosis treatment in routine clinical care, in which BMD and bone turnover markers were measured regularly as part of clinical follow-up. This cohort was approved by the institutional ethics committee (No. 17091229-5). Primary osteoporosis was diagnosed according to the Japanese diagnostic criteria based on the presence of vertebral or proximal femoral fragility fractures, other fragility fractures with a BMD below 80% of the young adult mean (YAM), or a lumbar spine or proximal femur BMD of ≤70% of YAM, corresponding approximately to a T-score of ≤−2.5 (Soen et al., 2013). Fracture history was collected from medical records, including clinical notes and radiographic reports. Patients were eligible if they had baseline DXA data and at least one follow-up DXA measurement within 3 years after denosumab initiation. Patients were excluded if baseline DXA data were unavailable, if no follow-up DXA data were available during the 3-year follow-up period, or if they had secondary osteoporosis, poorly controlled thyroid disease, active malignancy, or conditions that could substantially affect mobility or skeletal health, such as Parkinson’s disease. Because prior osteoporosis treatment may influence subsequent BMD response to denosumab, patients with no prior osteoporosis medication before denosumab initiation were defined as the treatment naive cohort and used for the primary analysis (Kendler et al., 2010; Miller et al., 2016; Tsuchiya et al., 2021). The overall cohort included patients regardless of prior osteoporosis treatment status and was analyzed as a secondary cohort.

### Data collection

Baseline demographic and clinical variables included age, sex, body mass index (BMI, kg/m^2^), smoking and alcohol. Laboratory variables assessed before treatment included serum calcium (mg/dL), phosphorus (mg/dL), 25-hydroxyvitamin D [25(OH)D; ng/mL], estimated glomerular filtration rate (eGFR, mL/min/1.73 m^2^) and alkaline phosphatase (ALP, U/L).

Serum bone turnover markers (BTMs) included tartrate-resistant acid phosphatase 5 b (TRACP-5b; reference range in women, 120–420 mU/dL; measured using the Osteolinks® TRACP-5b® test kit, DS Pharma Biomedical Co., Ltd., Osaka, Japan) and total N-terminal propeptide of type I procollagen (total P1NP; reference range in postmenopausal women, 26.4–98.2 μg/L; measured using a total P1NP assay on an Elecsys automated analyzer, Roche Diagnostics, Switzerland). BTMs were assessed at baseline and repeatedly during follow-up, including 6 months, 12 months, 24 months, and 36 months after denosumab initiation.

Dual-energy X-ray absorptiometry (DXA) of the lumbar spine and total hip was performed at baseline and during routine clinical follow-up, generally at 6- to 12-month intervals after denosumab initiation. Areal BMD of the lumbar spine (L1–L4) and total hip was measured using DXA (Hologic QDR series; Hologic, Waltham, MA, United States). All DXA measurements were analyzed centrally by a radiologist. The primary longitudinal outcome was percent BMD gain from baseline (%BMD gain), calculated as 100 × (follow-up BMD − baseline BMD)/baseline BMD.

### Statistical analysis

All statistical analyses were performed using R software (version 4.4.3; R Core Team, Vienna, Austria). Continuous variables were summarized as means with standard deviations, and categorical variables were summarized as counts and percentages. A two-sided p value <0.05 was considered statistically significant. Regression coefficients are reported with 95% confidence intervals and p values. Univariate comparisons across age groups were performed using one-way ANOVA for continuous variables and chi-square tests for categorical variables. For variables with significant overall differences, *post hoc* pairwise comparisons were performed using pairwise tests with Holm adjustment for multiple comparisons.

Multivariable linear mixed-effects models were used to examine the association between baseline age as a continuous variable and %BMD gain at 1 and 3 years after denosumab initiation. Follow-up time was modeled using natural cubic splines with 3 degrees of freedom, selected to allow nonlinear longitudinal changes in %BMD gain while maintaining model parsimony. Internal knots were placed at quantiles of the observed follow-up time, and boundary knots were placed at the minimum and maximum observed follow-up times. An interaction term between baseline age and follow-up time was included to estimate time-specific associations between age and %BMD gain. Models were constructed separately for lumbar spine BMD and total hip BMD. Covariates included sex, BMI, eGFR, and prior osteoporosis treatment status in the overall cohort. The overall age-by-time interaction was evaluated using Type III analysis of variance with Satterthwaite’s approximation for degrees of freedom. In the treatment naive cohort, prior osteoporosis treatment status was not included as a covariate. Models included patient-specific random intercepts and random slopes for follow-up time. Model convergence was assessed using convergence warnings from the model-fitting procedure and singular-fit diagnostics. Model diagnostics were performed by visually inspecting residual-versus-fitted plots and quantile–quantile plots of residuals. Model-based estimates were used to calculate the adjusted difference in %BMD gain per 1-year increase in baseline age at 1 and 3 years. Sensitivity analyses were performed with additional adjustment for baseline BMD and other clinically relevant covariates. To complement the above analysis, patients were categorized into age groups based on cohort specific quartiles: ≤72, 72 to 77, 77 to 83, and ≥83 years. Age group specific trajectories of %BMD gain through 3 years were modeled using natural spline based linear mixed effects models, providing model-based visualization of longitudinal BMD response patterns across age groups. Bone turnover markers were also summarized descriptively by age group at baseline, 6 months, and 1, 2, and 3 years after denosumab initiation, without formal hypothesis testing.

To assess potential selection bias, baseline characteristics were compared between treatment naive patients included in the longitudinal analysis and those excluded because of unavailable follow-up lumbar spine DXA measurements. Supplementary logistic regression analyses were performed to examine whether follow-up DXA availability was associated with observed baseline variables. Mixed-effects models used all available repeated measurements without imputation and were interpreted under a missing-at-random assumption, in which missing outcome data were assumed to depend on observed baseline characteristics and available follow-up measurements rather than on unobserved BMD outcomes.

## Results

### Baseline patient characteristics

**FIGURE 1:**
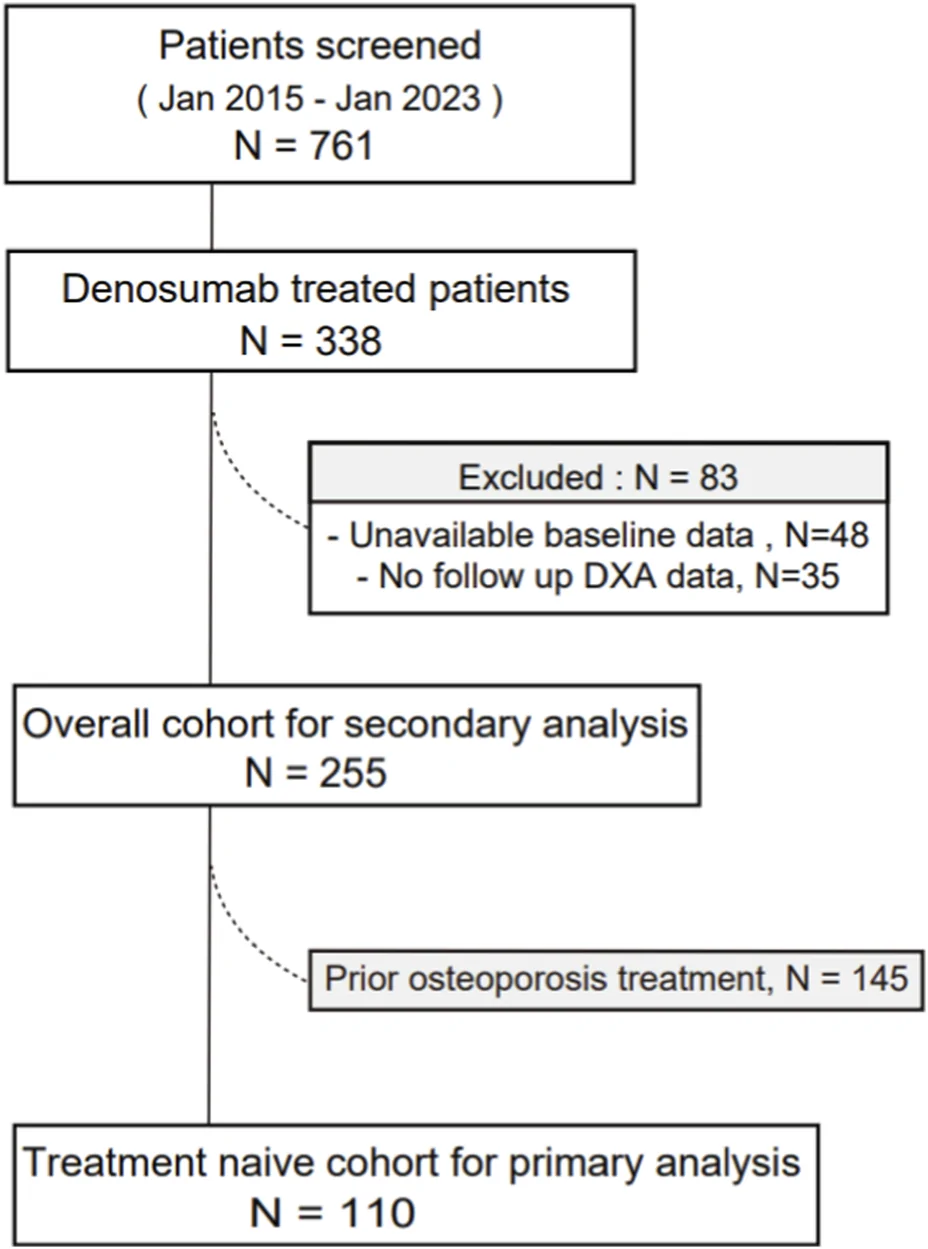
Patient flow diagram. Patients who initiated osteoporosis treatment between January 2015 and January 2023 were screened. Among 761 screened patients, 338 patients treated with denosumab were assessed for eligibility. Eighty-three patients were excluded because of unavailable baseline data or no follow up DXA data. The remaining 255 patients were included in the overall cohort for secondary analysis, and 110 treatment naive patients were included in the primary analysis.

Between January 2015 and January 2023, 761 patients who initiated osteoporosis treatment were screened. Of these, 338 patients treated with denosumab were assessed for eligibility, and 83 were excluded, including 48 with unavailable baseline data and 35 with no follow up DXA data during the 3 years follow up period. The remaining 255 patients were included in the overall cohort for secondary analysis. Among 125 treatment naive patients with available baseline lumbar spine BMD, 110 had at least one follow-up lumbar spine DXA measurement within 3 years and were included in the primary analysis (Figure 1).

In the treatment naïve patients, there were no significant differences in baseline characteristics across age groups, except for total hip BMD (Table 1). Post-hoc pairwise comparisons were performed for variables that showed significant overall differences across age groups. Baseline total hip BMD and T-scores differed mainly between the ≥83-year group and the younger age groups, particularly the ≤72-year and 72–77-year groups (both p < 0.001). In the overall cohort, although prior osteoporosis treatment did not differ significantly across age groups (p = 0.30), eGFR was lower in the older age groups (p < 0.001). Spine BMD was similar among age groups, whereas total hip BMD differed significantly across age groups and was lowest in the oldest group (≥83 years, 0.558 g/cm^2^; p < 0.001) (Supplementary Table S1). All but five patients received supplemental vitamin D to prevent hypocalcemia (Ishikawa et al., 2016).

**TABLE 1:** Demographic data in treatment naïve cohort.

Characteristic	Overall	≤72	72–77	77–83	≥83	p
n	110	36	24	27	23	​
Age, year (mean (SD))	76.3 (8.3)	67.4 (5.3)	74.5 (1.5)	80.1 (1.8)	87.6 (3.0)	<0.001
Male, n(%)	10 (9.1)	3 (8.3)	4 (16.7)	2 (7.4)	1 (4.3)	0.492
BMI, kg/m^2^ (mean (SD))	22.3 (4.7)	23.6 (4.8)	21.4 (6.4)	21.8 (3.6)	22.0 (3.6)	0.438
Smoking, n(%)	2 (1.8)	1 (2.9)	1 (4.2)	0 (0.0)	0 (0.0)	0.642
Alcohol, n(%)	2 (1.8)	0 (0.0)	1 (4.2)	1 (3.7)	0 (0.0)	0.503
Blood test						
eGFR, mL/min/1.73m^2^ (mean (SD))	65.1 (19.4)	71.4 (18.3)	65.5 (12.6)	61.0 (16.7)	59.6 (26.9)	0.073
Adjusted Ca, mg/dL (mean (SD))	9.3 (0.4)	9.4 (0.3)	9.2 (0.3)	9.2 (0.5)	9.3 (0.5)	0.324
IP, mg/dL (mean (SD))	3.6 (0.4)	3.7 (0.5)	3.6 (0.4)	3.5 (0.3)	3.5 (0.5)	0.469
ALP, U/L (mean (SD))	297.8 (105.3)	306.0 (100.5)	292.5 (83.5)	301.4 (125.1)	286.2 (112.9)	0.901
TRACP-5b, mU/dL (mean (SD))	537.0 (197.1)	500.1 (165.8)	556.5 (191.2)	557.0 (238.8)	552.0 (198.9)	0.599
Total P1NP, μg/L (mean (SD))	83.5 (66.1)	71.0 (32.6)	72.9 (41.4)	98.1 (78.8)	96.5 (99.6)	0.254
DXA measurement						
Spine - BMD, g/cm^2^ (mean (SD))	0.750 (0.155)	0.735 (0.152)	0.731 (0.117)	0.771 (0.174)	0.770 (0.176)	0.673
Spine - tscore (mean (SD))	−2.125 (1.386)	−2.261 (1.361)	−2.304 (1.038)	−1.930 (1.546)	−1.957 (1.572)	0.659
Total hip - BMD, g/cm^2^ (mean (SD))	0.623 (0.122)	0.654 (0.101)	0.673 (0.100)	0.594 (0.133)	0.551 (0.128)	<0.001
Total hip - tscore (mean (SD))	−2.526 (1.221)	−2.206 (1.012)	−2.054 (1.026)	−2.804 (1.319)	−3.236 (1.270)	<0.001

TRACP-5b = Tartrate-resistant acid phosphatase-5b; P1NP, procollagen type 1 N-terminal propeptide; IP, inorganic phosphate; ALP, alkaline phosphatase; Adjusted Ca = albumin-corrected calcium; DXA, Dual-energy X-ray absorptiometry; BMD, bone mineral density.

Post-hoc pairwise comparisons were performed only for variables with significant overall differences across age groups.

The number of patients assessed at each follow-up time point is summarized in Supplementary Table S2. The specific reasons for missing DXA measurements were not systematically recorded. Patients excluded because of unavailable follow-up DXA tended to be older than included patients, but the difference was not statistically significant (80.7 vs. 76.3 years, p = 0.063), and no other baseline demographic or clinical characteristic differed significantly between groups. Missingness analyses showed no significant association between follow-up DXA availability and age, baseline lumbar spine BMD, baseline lumbar spine T-score, eGFR, TRACP-5b, or P1NP.

### Longitudinal changes in bone turnover markers

TRACP 5 b and P1NP showed marked early suppression after denosumab initiation, and this suppression was generally maintained during follow up across age groups. Similar patterns were observed in the treatment naive cohort (Supplementary Figures S1a, b) and the overall cohort (Supplementary Figures S1c, d), with no apparent age dependent difference. These descriptive findings indicate that denosumab induced suppression of bone turnover markers was broadly comparable across age groups.

### Association between baseline age and lumbar spine BMD response

**FIGURE 2:**
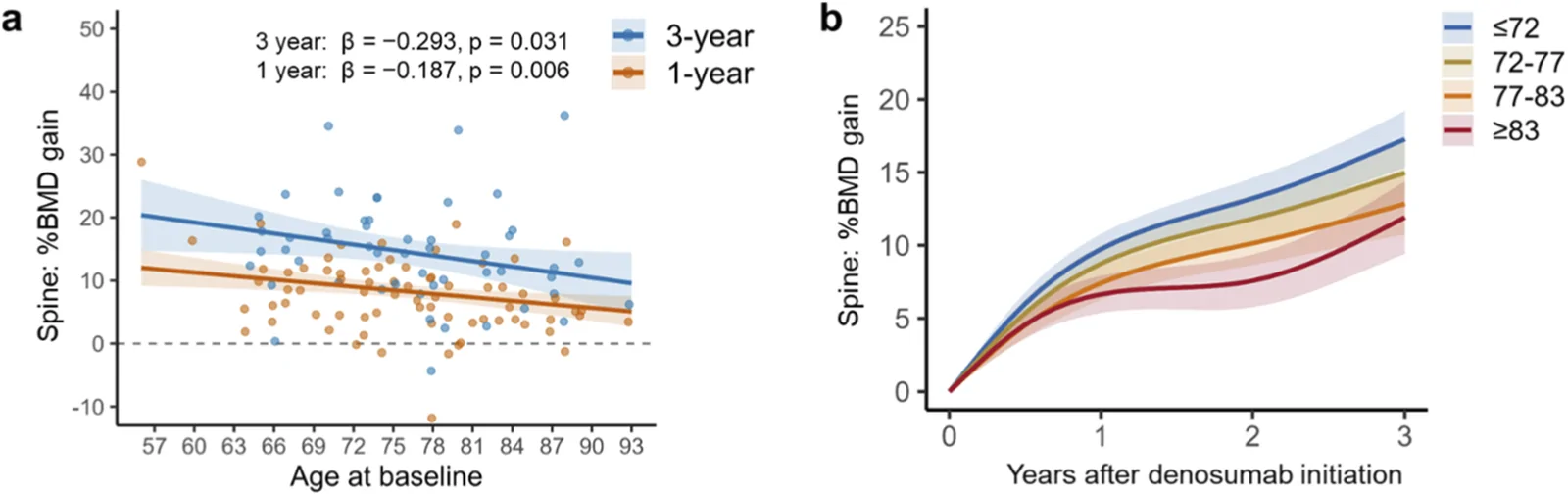
Association between baseline age and lumbar spine BMD response in the treatment naive cohort **(a)** Association between baseline age and lumbar spine %BMD gain at 1 and 3 years after denosumab initiation. Dots represent individual patients, and lines indicate model based estimates from multivariable linear mixed effects models. β values indicate the adjusted difference in %BMD gain per 1 year increase in baseline age. **(b)** Model based trajectories of lumbar spine %BMD gain through 3 years after denosumab initiation by age group. Patients were stratified into four age groups: ≤72, 72 to 77, 77 to 83, and ≥83 years. Shaded areas indicate 95% confidence intervals.

In the treatment naive cohort, the multivariable model adjusted for sex, BMI, and eGFR showed that higher baseline age was significantly associated with lower lumbar spine %BMD gain after denosumab treatment (overall age by time interaction, p = 0.013; Figure 2a). Model based estimates showed that older baseline age was associated with lower lumbar spine %BMD gain, with a 0.187 percentage point lower gain at 1 year and a 0.293 percentage point lower gain at 3 years for each additional year of age (1 year: β = −0.187, p = 0.006; 3 years: β = −0.293, p = 0.031; Supplementary Table S3). Clinically, these estimates translate to approximately 1.9 and 2.9 percentage-point lower lumbar spine %BMD gains at 1 and 3 years, respectively, for each 10-year increase in baseline age. In the overall cohort, baseline age was not significantly associated with lumbar spine %BMD gain at either 1 or 3 years (Supplementary Figure S2a; Supplementary Table S3). Sensitivity analyses additionally adjusting for baseline lumbar spine BMD, as well as expanded models including prevalent fracture history, vitamin D supplementation, comorbidities, and baseline bone turnover markers, showed generally consistent findings (Supplementary Table S4).

To complement the continuous age analysis, patients were divided into four age groups based on cohort specific quartiles of baseline age. Model based trajectories showed that lumbar spine %BMD gain increased over time across all age groups in both the treatment naive cohort and the overall cohort. In the treatment naive cohort, the youngest age group showed numerically greater 3 years %BMD gain than the oldest age group (≤72 years: 17.8% versus ≥83 years: 12.6%; Figure 2b). In the overall cohort, the age group trajectories were more closely aligned, with no clear age dependent separation (Supplementary Figure S2b). These findings were consistent with the continuous age analysis and illustrated longitudinal response patterns across age groups.

### Association between baseline age and total hip BMD response

**FIGURE 3:**
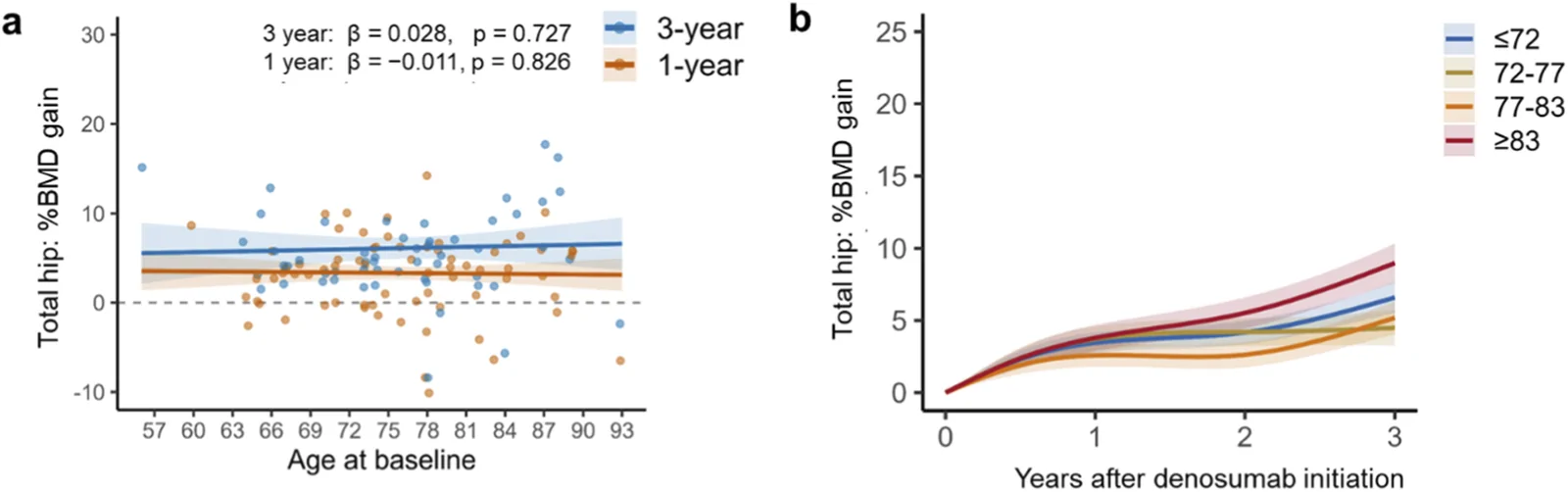
Association between baseline age and total hip BMD response in the treatment naive cohort **(a)** Association between baseline age and total hip %BMD gain at 1 and 3 years after denosumab initiation. Dots represent individual patients, and lines indicate model based estimates from multivariable linear mixed effects models. β values indicate the adjusted difference in %BMD gain per 1 year increase in baseline age. **(b)** Model based trajectories of total hip %BMD gain through 3 years after denosumab initiation by age group. Patients were stratified into four age groups: ≤72, 72 to 77, 77 to 83, and ≥83 years. Shaded areas indicate 95% confidence intervals.

In the treatment naive cohort, baseline age was not significantly associated with total hip %BMD gain at 1 or 3 years after denosumab initiation (1 year: β = −0.011, p = 0.826; 3 years: β = 0.028, p = 0.727; Figure 3a; Supplementary Table S5). Consistent with this, model-based age group trajectories showed longitudinal increases in total hip %BMD gain across age groups, without a clear pattern of attenuated response in older patients (Figure 3b).

In the overall cohort, time point specific age trends at 1 and 3 years were not statistically significant (1 year: β = −0.008, p = 0.787; 3 years: β = 0.127, p = 0.052; Supplementary Figure S3b; Supplementary Table S5). Age group trajectories in the overall cohort also showed no clear age dependent separation (Supplementary Figure S3b). Sensitivity analyses additionally adjusting for baseline total hip BMD and expanded clinical covariates showed generally consistent findings, with no significant association between baseline age and total hip %BMD gain in the treatment-naive cohort (Supplementary Table S6). Overall, no statistically significant association was observed between baseline age and total hip %BMD gain in either cohort.

## Discussion

The main finding of this study was that older patient age was associated with a modestly smaller lumbar spine %BMD gain after denosumab treatment in treatment naive patients, whereas no significant age related association was observed at the total hip. This association was not evident in the overall cohort, suggesting that the influence of age on denosumab associated BMD gain is limited, but may be detectable at the lumbar spine in patients without prior osteoporosis treatment.

Previous studies have supported the effectiveness of denosumab in older patients with osteoporosis. In subgroup analyses of the FREEDOM trial, denosumab reduced fracture risk across age-related subgroups, including women aged 75 years or older (McClung et al., 2012). Real-world studies of patients aged 80 years or older have also reported significant BMD gains after denosumab treatment (Jeong and Ha, 2020). In the present relatively older cohort, no statistically significant age-related association was observed for total hip %BMD gain, whereas lumbar spine %BMD gain was modestly smaller in older treatment-naive patients.

The observed association between older age and smaller lumbar spine %BMD gain in treatment naive patients may reflect age related changes in skeletal remodeling and bone formation capacity (Khosla, 2013; Raisz, 2005; Marie and Kassem, 2011). The magnitude of BMD gain may depend on baseline remodeling activity and the capacity of the bone microenvironment to respond over time. Aging is associated with reduced osteoblast function, altered coupling between resorption and formation, and changes in marrow and skeletal microarchitecture (Marie and Kassem, 2011; Weivoda et al., 2020; Piemontese et al., 2017; Stenderup et al., 2003; Zhou et al., 2008). These age-related changes could blunt the apparent gain in BMD at the lumbar spine, even when bone resorption is effectively suppressed. In our cohort, both TRACP-5b and P1NP decreased after denosumab initiation across age groups. Thus, the modestly smaller lumbar spine response in older patients is unlikely to be explained solely by inadequate suppression of bone turnover, as reflected by these markers.

The site-specific pattern of our findings warrants consideration. Older age was associated with a smaller lumbar spine %BMD gain in treatment-naive patients, whereas no statistically significant age-related association was observed for total hip %BMD gain. This difference may partly reflect the effects of denosumab on cortical bone, including suppression of intracortical remodeling and reduction of cortical porosity (Zebaze et al., 2016; Zebaze et al., 2014). Unlike bisphosphonates, denosumab does not bind to bone and may more readily access intracortical remodeling spaces, which may contribute to its cortical effects (Baron et al., 2011). In addition, the lower baseline total hip BMD observed in the oldest patients may have contributed to the results.

An important observation was that the age-related association seen in the treatment naive cohort was not evident in the overall cohort. Prior osteoporosis treatment may have modified subsequent BMD response to denosumab and obscured age-related differences. Patients who previously received osteoporosis medications may start denosumab with different bone remodeling states and residual drug effects from prior therapy (Tsuchiya et al., 2021). These factors can influence the magnitude and timing of BMD change after switching to denosumab. Therefore, the treatment naive cohort may provide a clearer assessment of the relationship between age and the initial BMD response to denosumab. By contrast, the overall cohort may better reflect real world clinical practice, where patients often receive denosumab after other osteoporosis medications.

This study has several strengths. We evaluated longitudinal BMD responses for up to 3 years, which provides relatively longer treatment among real-world studies. We also analyzed baseline age as a continuous variable using mixed effects models, while complementing this approach with age group-based trajectory analyses. In addition, we performed separate analyses in treatment naive patients and in the overall cohort, allowing us to distinguish a cleaner initial treatment response from a broader real-world population with prior medication exposure.

Several limitations should be acknowledged. First, this was a retrospective observational study. Second, the sample size was modest, particularly after stratification by age group and treatment history. Third, BMD was assessed using DXA, which does not capture bone microarchitecture or bone strength and may be affected by osteophytes and vascular calcification (Ishikawa et al., 2024; Keaveny et al., 2025). Fourth, data on the duration of prior osteoporosis treatment and exact denosumab dosing intervals were not available. Fifth, fracture outcomes were not evaluated as a primary endpoint due to the limited sample size. Finally, safety parameters and adverse events were not systematically collected.

In conclusion, baseline age showed a modest, site-specific association with denosumab-related %BMD gain. In treatment-naive patients, older age was associated with smaller lumbar spine %BMD gain, whereas no statistically significant age-related association was observed for total hip %BMD gain. These findings suggest that denosumab-related BMD responses may vary by skeletal site in treatment-naive patients. Together, these results may help interpret denosumab-related BMD responses in older patients.

## Data Availability

The original contributions presented in the study are included in the article/Supplementary Material, further inquiries can be directed to the corresponding author.
