# Supplementary material for: Age-related differences in BMD response during three years of denosumab treatment
Source: Front Aging. 2026 Aug 13;7:1892852. doi: 10.3389/fragi.2026.1892852 (PMC13518595; doi:10.3389/fragi.2026.1892852)
Supplement: Supplementary file 1 [file Supplementaryfile1.docx]

**Supplementary file**

Supplementary Figure S1-3

Supplementary Table 1-6

**Age-Related Differences in BMD Response During Three Years of Denosumab Treatment**

Koji Ishikawa^1^, Tomoyuki Asada^2^, William Richardson^1^, Choiselle Marius^1^, Mahoko Ishikawa^1^, Tuyet Nguyen^1^, Philip Varnadore^1^, Soji Tani^3^, Peter Passias^1^, Benjamin Aaron Alman^1^

1) Department of Orthopaedic Surgery, Duke University, NC, USA
2) Department of Orthopedic Surgery, Tsukuba Memorial Hospital, Ibaraki, Japan
3) Department of Orthopaedic Surgery, Showa Medical University, Tokyo, Japan

*Corresponding author: Koji Ishikawa (K.I)

Department of Orthopaedics, Duke University

Address: 308 Research Drive, LSRC B330A, Durham, NC 27710, USA

Tel: 919-681-1797

Email: [koji.ishikawa@duke.edu](mailto:koji.ishikawa@duke.edu)

**Supplementary Figure S1. Changes in bone turnover markers after denosumab initiation by age group**

a, Longitudinal changes in serum TRACP-5b levels in the treatment naive subset. b, Longitudinal changes in serum P1NP levels in the treatment naive subset. c, Longitudinal changes in serum TRACP-5b levels in the overall cohort. d, Longitudinal changes in serum P1NP levels in the overall cohort. Patients were stratified into four age groups: ≤72, 72 to 77, 77 to 83, and ≥83 years. Values are shown at baseline and 0.5, 1, 2, and 3 years after denosumab initiation. Lines and points indicate mean values, and shaded areas indicate SD.

**Supplementary Figure S2. Association between baseline age and lumbar spine BMD response in the overall cohort**

a, Association between baseline age and lumbar spine %BMD gain at 1 and 3 years after denosumab initiation in the overall cohort. Dots represent individual patients, and lines indicate model based estimates from multivariable linear mixed effects models. β values indicate the adjusted difference in %BMD gain per 1 year increase in baseline age.
b, Model based trajectories of lumbar spine %BMD gain through 3 years after denosumab initiation by age group in the overall cohort. Patients were stratified into four age groups: ≤72, 72 to 77, 77 to 83, and ≥83 years. Shaded areas indicate 95% confidence intervals.

**Supplementary Figure S3. Association between baseline age and total hip BMD response in the overall cohort**

a, Association between baseline age and total hip %BMD gain at 1 and 3 years after denosumab initiation in the overall cohort. Dots represent individual patients, and lines indicate model based estimates from multivariable linear mixed effects models. β values indicate the adjusted difference in %BMD gain per 1 year increase in baseline age.
b, Model based trajectories of total hip %BMD gain through 3 years after denosumab initiation by age group in the overall cohort. Patients were stratified into four age groups: ≤72, 72 to 77, 77 to 83, and ≥83 years. Shaded areas indicate 95% confidence intervals.

| **Characteristic** | **Overall** | **≤ 72** | **72-77** | **77-83** | **≥ 83** | **p** |
| --- | --- | --- | --- | --- | --- | --- |
| **n** | 255 | 74 | 56 | 65 | 60 |  |
| **Age, year (mean (SD))** | 77.0 (8.2) | 67.2 (4.8) | 74.8 (1.4) | 80.3 (1.7) | 87.4 (2.8) | <0.001 |
| **Male, n (%)** | 16 (6.3) | 5 (6.8) | 6 (10.7) | 4 (6.2) | 1 (1.7) | 0.254 |
| **BMI, kg/m^2^ (mean (SD))** | 21.8 (3.9) | 21.8 (4.2) | 21.9 (4.3) | 22.0 (3.3) | 21.4 (3.9) | 0.926 |
| **Smoking, n (%)** | 11 (4.3) | 5 (6.8) | 2 (3.6) | 2 (3.1) | 2 (3.3) | 0.758 |
| **Alcohol, n (%)** | 7 (2.7) | 1 (1.4) | 3 (5.4) | 3 (4.6) | 0 (0.0) | 0.226 |
| **Prior treatment** |  | | | | | |
| **Bisphosphonate** | 42 (16.5) | 9 (12.2) | 8 (14.3) | 10 (15.4) | 15 (25.0) |  |
| **PTH** | 42 (16.5) | 8 (10.8) | 11 (19.6) | 15 (23.1) | 8 (14.3) |  |
| **Romosozumab** | 48 (18.8) | 15 (20.3) | 12 (21.4) | 11 (16.9) | 10 (16.7) |  |
| **SERM** | 5 (2.2) | 3 (4.1) | 0 (0.0) | 2 (3.1) | 0 (0.0) |  |
| **Blood test** |  | | | | | |
| **eGFR, mL/min/1.73m² (mean (SD))** | 64.5 (18.2) | 70.3 (14.9) | 66.7 (17.2) | 63.1 (16.4) | 56.7 (21.7) | <0.001 |
| **Adjusted Ca, mg/dL (mean (SD))** | 9.4 (0.4) | 9.4 (0.4) | 9.3 (0.4) | 9.3 (0.4) | 9.3 (0.5) | 0.871 |
| **IP, mg/dL (mean (SD))** | 3.5 (0.5) | 3.5 (0.5) | 3.6 (0.4) | 3.6 (0.4) | 3.5 (0.5) | 0.662 |
| **ALP, U/L (mean (SD))** | 247.3 (118.1) | 264.2 (113.7) | 226.3 (112.7) | 238.9 (125.4) | 254.8 (119.7) | 0.28 |
| **TRACP-5b, mU/dL (mean (SD))** | 436.2 (222.8) | 401.0 (190.3) | 436.0 (223.7) | 462.2 (246.5) | 452.5 (232.8) | 0.388 |
| **Total P1NP, μg/L (mean (SD))** | 66.6 (53.7) | 58.6 (33.8) | 60.5 (36.9) | 71.7 (61.6) | 77.0 (74.0) | 0.168 |
| **DXA measurement** |  | | | | | |
| **Spine - BMD, g/cm² (mean (SD))** | 0.772 (0.162) | 0.741 (0.135) | 0.809 (0.169) | 0.786 (0.166) | 0.759 (0.176) | 0.084 |
| **Spine - Tscore (mean (SD))** | -1.933 (1.444) | -2.209 (1.209) | -1.609 (1.514) | -1.791 (1.463) | -2.050 (1.574) | 0.087 |
| **Total hip - BMD, g/cm² (mean (SD))** | 0.623 (0.113) | 0.655 (0.089) | 0.670 (0.090) | 0.606 (0.116) | 0.558 (0.122) | <0.001 |
| **Total hip - Tscore (mean (SD))** | -2.521 (1.129) | -2.195 (0.899) | -2.064 (0.913) | -2.686 (1.153) | -3.178 (1.215) | <0.001 |

**Supplementary Table 1. Demographic Data in overall cohort**

PTH = parathyroid hormone analog; SERM = selective estrogen receptor modulator; TRACP-5b = Tartrate-resistant acid phosphatase-5b; P1NP = procollagen type 1 N-terminal propeptide; IP = inorganic phosphate; ALP = Alkaline phosphatase; Adjusted Ca=albumin-corrected calcium; DXA = Dual-energy X-ray absorptiometry; BMD = bone mineral density

| Time point | n / total | % |
| --- | --- | --- |
| Baseline | 110/110 | 100 |
| 6 months | 108/110 | 98.2 |
| 12 months | 102/110 | 92.7 |
| 18 months | 59/110 | 53.6 |
| 24 months | 83/110 | 75.5 |
| 30 months | 40/110 | 36.4 |
| 36 months | 75/110 | 68.2 |

**Supplementary Table 2. Number of treatment-naive patients with lumbar spine DXA measurements at each follow-up time point.**

|  | **Treatment naive cohort** | | **Overall cohort** | |
| --- | --- | --- | --- | --- |
|  | **β (95% CI)** | **P value** | **β (95% CI)** | **P value** |
| **Year 1** | -0.187 [-0.320, -0.056] | 0.006 | 0.046 [-0.071, 0.164] | 0.439 |
| **Year 3** | -0.293 [-0.559, -0.027] | 0.031 | 0.027 [-0.153, 0.208] | 0.767 |

**Supplementary Table 3. Multivariable model results for lumbar spine %BMD gain at 1 and 3 years**

β values indicate the adjusted difference in lumbar spine %BMD gain per 1 year increase in baseline age. Models were adjusted for sex, BMI, and eGFR in the treatment naive cohort, and for sex, BMI, eGFR, and prior osteoporosis treatment status in the overall cohort. CI = confidence interval; BMD = bone mineral density; eGFR = estimated glomerular filtration rate.

|  | **Treatment naive cohort** | | **Overall cohort** | |
| --- | --- | --- | --- | --- |
|  | **β (95% CI)** | **P value** | **β (95% CI)** | **P value** |
| **Model 2** |  |  |  |  |
| **Year 1** | -0.156 [-0.277, -0.034] | 0.013 | 0.038 [-0.073, 0.149] | 0.500 |
| **Year 3** | -0.259 [-0.516, -0.002] | 0.048 | 0.020 [-0.153, 0.193] | 0.819 |
| **Model 3** |  |  |  |  |
| **Year 1** | -0.184 [-0.296, -0.071] | 0.002 | -0.023 [-0.142, 0.096] | 0.702 |
| **Year 3** | -0.289 [-0.543, -0.036] | 0.026 | -0.022 [-0.210, 0.166] | 0.818 |

**Supplementary Table 4. Sensitivity multivariable model for lumbar spine %BMD gain at 1 and 3 years**

β values indicate the adjusted difference in lumbar spine %BMD gain per 1 year increase in baseline age. Model 2 was adjusted for sex, BMI, eGFR, and baseline lumbar spine BMD in the treatment-naive cohort, and additionally adjusted for prior osteoporosis treatment status in the overall cohort. Model 3 was further adjusted for prevalent fracture history, vitamin D supplementation, steroid use, rheumatoid arthritis, smoking, alcohol, TRACP-5b, and P1NP. CI = confidence interval; BMD = bone mineral density; eGFR = estimated glomerular filtration rate; TRACP-5b = tartrate-resistant acid phosphatase 5b; P1NP = N-terminal propeptide of type I procollagen.

|  | **Treatment naive cohort** | | **Overall cohort** | |
| --- | --- | --- | --- | --- |
|  | **β (95% CI)** | **P value** | **β (95% CI)** | **P value** |
| **Year 1** | -0.011 [-0.113, 0.090] | 0.826 | -0.008 [-0.070, 0.053] | 0.787 |
| **Year 3** | 0.028 [-0.133, 0.190] | 0.727 | 0.127 [-0.001, 0.255] | 0.052 |

**Supplementary Table 5. Multivariable model results for total hip %BMD gain at 1 and 3 years**

β values indicate the adjusted difference in total hip %BMD gain per 1 year increase in baseline age. Models were adjusted for sex, BMI, and eGFR in the treatment naive cohort, and for sex, BMI, eGFR, and prior osteoporosis treatment status in the overall cohort. CI = confidence interval; BMD = bone mineral density; eGFR = estimated glomerular filtration rate.

|  | **Treatment naive cohort** | | **Overall cohort** | |
| --- | --- | --- | --- | --- |
|  | **β (95% CI)** | **P value** | **β (95% CI)** | **P value** |
| **Model 2** |  |  |  |  |
| **Year 1** | -0.030 [-0.135, 0.074] | 0.566 | -0.022 [-0.086, 0.042] | 0.499 |
| **Year 3** | 0.009 [-0.152, 0.170] | 0.912 | 0.113 [-0.016, 0.241] | 0.085 |
| **Model 3** |  |  |  |  |
| **Year 1** | -0.040 [-0.128, 0.047] | 0.364 | -0.038 [-0.101, 0.026] | 0.246 |
| **Year 3** | -0.000 [-0.154, 0.154] | 0.999 | 0.136 [0.001, 0.271] | 0.048 |

**Supplementary Table 6. Sensitivity multivariable model for total hip %BMD gain at 1 and 3 years**

β values indicate the adjusted difference in total hip %BMD gain per 1 year increase in baseline age. Model 2 was adjusted for sex, BMI, eGFR, and baseline total hip BMD in the treatment-naive cohort, and additionally adjusted for prior osteoporosis treatment status in the overall cohort. Model 3 was further adjusted for prevalent fracture history, vitamin D supplementation, steroid use, rheumatoid arthritis, smoking, alcohol, TRACP-5b, and P1NP. CI = confidence interval; BMD = bone mineral density; eGFR = estimated glomerular filtration rate; TRACP-5b = tartrate-resistant acid phosphatase 5b; P1NP = N-terminal propeptide of type I procollagen.
